# A hypomorphic mutation in *Pold1* disrupts the coordination of embryo size expansion and morphogenesis during gastrulation

**DOI:** 10.1242/bio.059307

**Published:** 2022-08-08

**Authors:** Tingxu Chen, Heather Alcorn, Sujan Devbhandari, Dirk Remus, Elizabeth Lacy, Danwei Huangfu, Kathryn V. Anderson

**Affiliations:** 1 Developmental Biology Program, Sloan Kettering Institute, Memorial Sloan Kettering Cancer Center, New York, NY 10065, USA; 2 Louis V. Gerstner Jr. Graduate School of Biomedical Sciences, Memorial Sloan Kettering Cancer Center, New York, NY 10065, USA; 3 Molecular Biology Program, Sloan Kettering Institute, Memorial Sloan Kettering Cancer Center, New York, NY 10065, USA

**Keywords:** *Pold1*, Cell proliferation, Gastrulation, Embryo size, Morphogenesis, Lineage specification

## Abstract

Formation of a properly sized and patterned embryo during gastrulation requires a well-coordinated interplay between cell proliferation, lineage specification and tissue morphogenesis. Following transient physical or pharmacological manipulations of embryo size, pre-gastrulation mouse embryos show remarkable plasticity to recover and resume normal development. However, it remains unclear how mechanisms driving lineage specification and morphogenesis respond to defects in cell proliferation during and after gastrulation. Null mutations in DNA replication or cell-cycle-related genes frequently lead to cell-cycle arrest and reduced cell proliferation, resulting in developmental arrest before the onset of gastrulation; such early lethality precludes studies aiming to determine the impact of cell proliferation on lineage specification and morphogenesis during gastrulation. From an unbiased ENU mutagenesis screen, we discovered a mouse mutant, *tiny siren* (*tyrn*), that carries a hypomorphic mutation producing an aspartate to tyrosine (D939Y) substitution in Pold1, the catalytic subunit of DNA polymerase δ. Impaired cell proliferation in the *tyrn* mutant leaves anterior–posterior patterning unperturbed during gastrulation but results in reduced embryo size and severe morphogenetic defects. Our analyses show that the successful execution of morphogenetic events during gastrulation requires that lineage specification and the ordered production of differentiated cell types occur in concordance with embryonic growth.

## Abstract (precis)

**Summary:** A hypomorphic mutation in *Pold1* caused reduced size and abnormal morphology of gastrulating mouse embryos, highlighting the importance of coordinated embryo growth, lineage specification, and tissue morphogenesis for normal embryogenesis.

## INTRODUCTION

Gastrulation is a critical developmental process required for germ layer formation and the establishment of the body plan (Arnold and Robertson, 2009). Gastrulation initiates with the emergence of the primitive streak in the proximal posterior epiblast. As the streak extends to the distal tip of the embryo, epiblast cells undergo an epithelial-mesenchymal transition (EMT) to form the mesoderm layer between the epiblast and the visceral endoderm (VE) (Kinder et al., 1999; Lawson, 1999; Lawson et al., 1991). Epiblast cells ingressing through the anterior region of the elongating primitive streak intercalate into the VE to form the definitive endoderm layer, which will give rise to the gut tube, and subsequently, the epithelium of endodermal organs, such as the pancreas and intestine (Kwon et al., 2008; Lawson et al., 1986; Lawson and Pedersen, 1987). Gastrulation requires tight spatiotemporal coordination of cell number expansion, cell migration, and cell fate determination. Despite extensive research on these topics, it has been challenging to untangle the complex interplay among these three key components. Previous studies used embryological methods in pre-implantation embryos to investigate size regulation during the pre- and early-gastrulation stages. These experiments found that double-sized embryos, formed by aggregating two eight-cell-stage morula, underwent size regulation before gastrulation. The double-sized embryos showed an increase in cell-cycle length compared to controls; in addition, they lacked the proliferative burst that normally occurs before gastrulation. These two modes of regulating cell proliferation allowed the aggregated embryos to reach a normal size and cell number before E7.0 and then to gastrulate (Buehr and McLaren, 1974; Lewis and Rossant, 1982). Conversely, undersized mouse embryos generated by removing one or two blastomeres from the four-cell-stage preimplantation embryo, sustained a prolonged proliferative burst, leading to an increase in cell number before the initiation of gastrulation (Power and Tam, 1993). Another study on size regulation in the mammalian embryo examined the response to reduced cell numbers in the early post-implantation embryo, an experimentally more refractory stage. Following treatment with mitomycin to inhibit cell proliferation, E7.0 embryos, with ∼80% of their cells eliminated, could still recover and complete gastrulation (Snow and Tam, 1979). These elegant studies suggest that intrinsic mechanisms operate within the pre- and early-post-implantation embryo to monitor and control cell numbers both before and at the onset of gastrulation, supporting regulative development as an important feature of early mammalian embryogenesis. However, it is technically challenging to apply standard embryological and pharmacological methods to investigate how altered cell number in the gastrulating embryo impacts tissue patterning and morphogenesis. Therefore, to ask whether mechanisms of size regulation continue to act during gastrulation, it is beneficial to explore alternative approaches to perturbing cell numbers, such as genetic manipulation.

Although many genes encoding cell-cycle-related proteins have been genetically inactivated to explore the effects of cell proliferation on embryo size and morphogenesis, the resulting phenotypes are generally not suitable for studies of gastrulation. For example, *Cyclin A2* (*Ccna2*) homozygous null mutants can be recovered only up to E5.5 (Murphy et al., 1997), whereas embryos lacking all D-type cyclins survive past gastrulation, with no overt phenotypes (Kozar et al., 2004). Other targets for genetic perturbation of cell proliferation are the polymerases that replicate DNA. DNA Polymerase Delta (Pol δ), the subject of this report, plays multiple critical roles in DNA replication, with functions in DNA synthesis and repair (Jain et al., 2018). In mammalian cells, Pol δ contains four subunits: the catalytic subunit, p125 (Pold1), and three regulatory subunits, p50 (Pold2), p66 (Pold3) and p12 (Pold4). Pold1 consists of two functional domains: an N-terminal 3′-5′ exonuclease with DNA proofreading activity and a C-terminal DNA polymerase that catalyses DNA synthesis. Several mutant alleles have been generated for *Pold1* but, like the targeted cell-cycle-related genes mentioned above, the homozygous mutants either fail to survive beyond the onset of gastrulation or show no phenotypic defects during gastrulation. Null mutations in *Pold1* cause peri-implantation lethality (Uchimura et al., 2009). Two missense mutations have been reported for *Pold1*: a D400A substitution in the exonuclease domain and an L604K substitution in the DNA polymerase active site (Fig. S1A) (Uchimura et al., 2009; Venkatesan et al., 2007). While developmentally normal, *Pold1^D400A/D400A^* mice frequently died with swollen thymuses 3 months after birth (Uchimura et al., 2009). *Pold1^+/L604K^* heterozygous mice underwent normal development but had a reduced lifespan and developed multiple tumour types, including lymphoma, adenoma, and carcinomas of the liver and lung (Venkatesan et al., 2007). Although no specific phenotypes were reported for *Pold1^L604K/L604K^* embryos, notably they died around E8.5, suggesting that missense mutations in the polymerase domain might perturb cell proliferation at a level compatible with the investigation of gastrulation phenotypes.

In this study, we report a *Pold1* hypomorphic mutation identified in a phenotype-based genetic screen for recessive mutations causing gastrulation defects in mouse embryos. This mutation altered a conserved residue (D939Y) in the Pold1 DNA polymerase domain, caused reduced Pold1 protein expression, and resulted in compromised DNA synthesis. Mutant embryos could be retrieved up to E8.5; at this stage they were small with a siren-like morphology; hence we named the mutant *tiny siren* (*tyrn*). We investigated embryo growth and cell lineage differentiation in *tyrn* mutants at developmental stages between E6.5 and E8.5. The *tyrn* mutation impaired cell proliferation without affecting anterior–posterior (A–P) patterning, but severely disrupted tissue morphogenesis during gastrulation. Our findings suggest normal cell proliferation is essential for mesoderm lineage allocation and is required to coordinate embryo size with cell movement for proper morphogenesis.

## RESULTS

### *tyrn* mutant embryos show abnormal morphology but proper A–P patterning

**Fig. 1.**
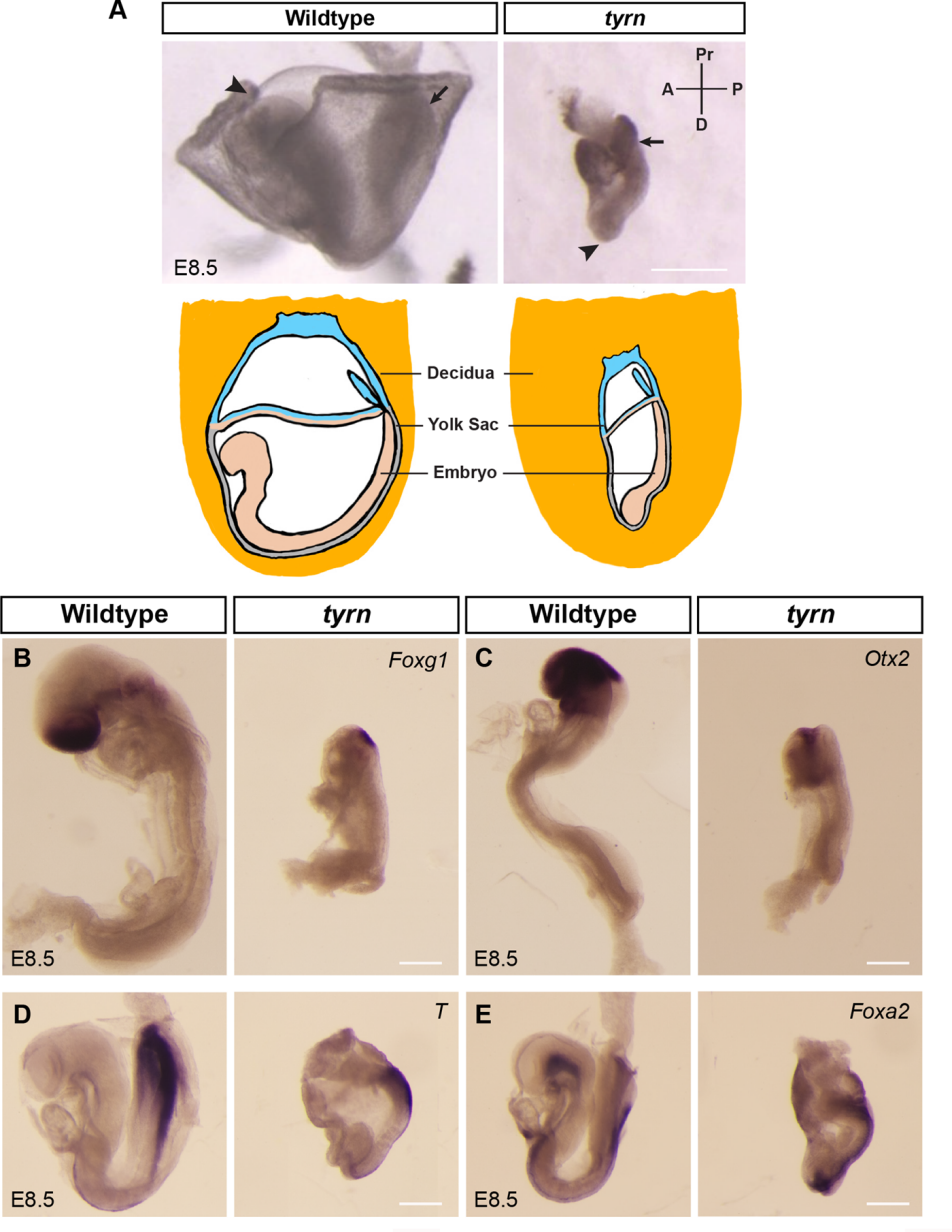
**Characterisation of *tiny siren* (*tyrn*), a mutant recovered from an ENU Screen.** (A) Top: wildtype and *tyrn* embryos recovered at E8.5. Embryos were aligned as they were in the decidua. Mutant embryos showed a shifted A–P body axis, with a small head located at the distal tip (arrowhead) and the tail at the posterior–proximal side (arrow). Scale bar: 500 μm. Bottom: cartoon showing the orientation of wildtype and mutant embryos inside the decidua. (B) *Foxg1* labels forebrain and (C) *Otx2* labels midbrain and forebrain in wildtype embryos. Mutant embryos expressed both *Foxg1* (B) and *Otx2* (C) in the head region. (D)*T* (*Brachyury*) marks the primitive streak and notochord in E8.5 wildtype and *tyrn* embryos. (E) Both wildtype and *tyrn* embryos expressed *Foxa2* in the floor plate, posterior notochord, notochordal plate and gut endoderm. *n*=3 embryos per genotype. Scale bar: 200 μm.

To study the genetic regulation of gastrulation, we performed mouse ENU mutagenesis screens to uncover genetic disruptions in embryos with abnormal morphology at E8.5 (García-García et al., 2005; Hernández-Martínez et al., 2019; Huangfu et al., 2003; Migeotte et al., 2011). We isolated a mutant (later named *tyrn*) that exhibited not only a smaller overall size, but also a striking body shape and orientation (Fig. 1A) that was distinct from other mutant phenotypes we had observed at this stage (Bazzi et al., 2017; García-García et al., 2005; Hernández-Martínez et al., 2019; Huangfu et al., 2003; Migeotte et al., 2011; Zhou and Anderson, 2010). Instead of forming a U-shaped embryo with a well-extended A–P axis, the *tyrn* mutant embryos had a short A–P axis and lay relatively flat along the posterior side of the yolk sac, with the head misoriented towards the distal tip (Fig. 1A). To determine if the irregularly shaped mutants had established the A–P axis, we performed whole-mount *in situ* hybridisations (WISH) at E8.5 to a diagnostic set of markers. We found that although *tyrn* mutant embryos did not form a well-structured head, they did express *Foxg1* and *Otx2*, markers of forebrain and forebrain/midbrain, respectively, in discrete overlapping regions (Fig. 1B,C). In addition, WISH detected relatively normal expression of *T* (*Brachyury*), which marks the posterior tail bud and notochord (Fig. 1D), and of *Foxa2*, which labels the floor plate of the neural tube, posterior notochord, notochordal plate and gut endoderm (Fig. 1E). Therefore, despite causing a highly unusual body shape, the *tyrn* mutation does not affect A–P patterning.

### *tyrn* is a hypomorphic allele of *Pold1*

**Fig. 2.**
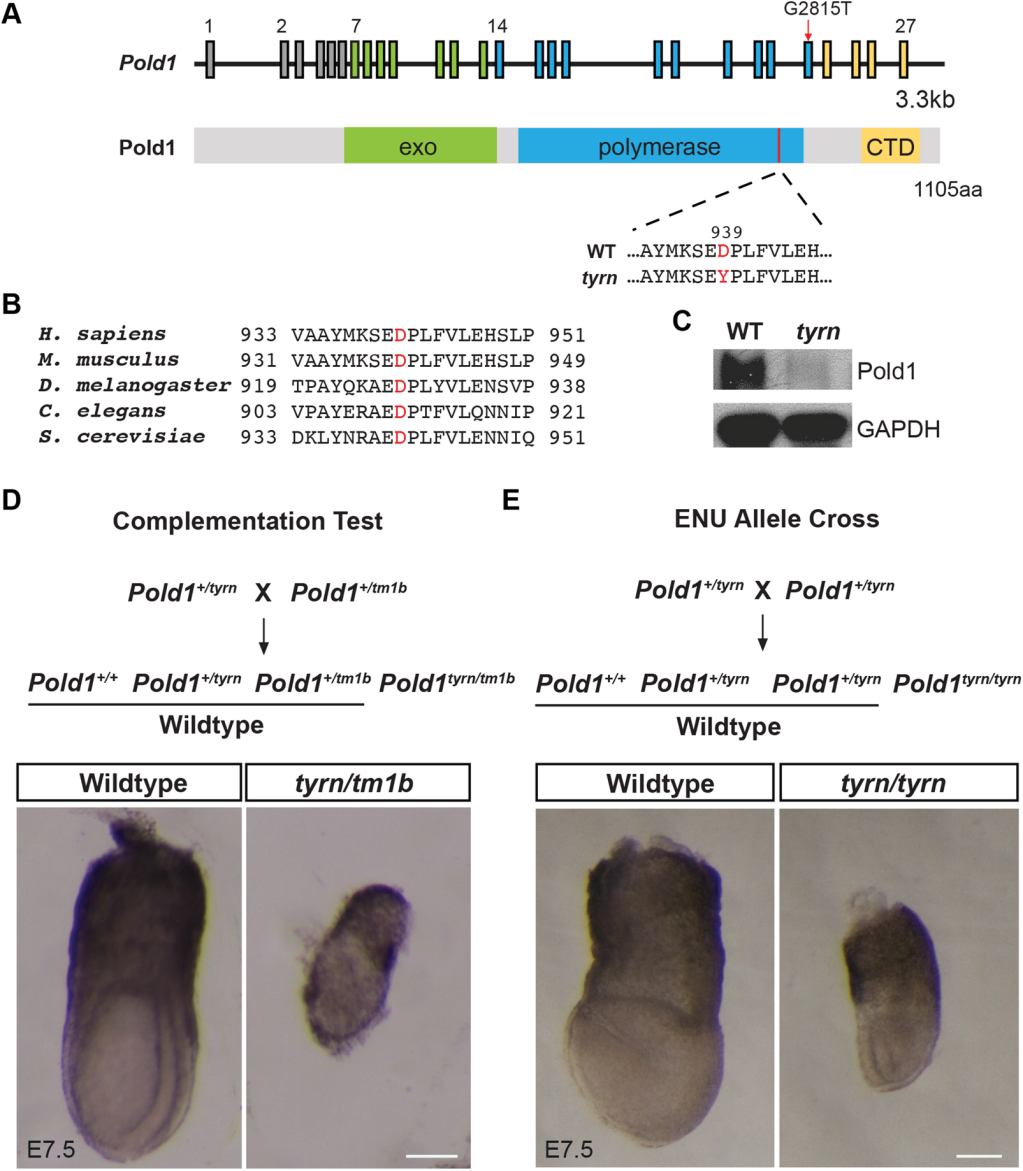
**Identification of the *Pold1* missense mutation in *tyrn*.** (A) Schematic diagrams of the murine *Pold1* genomic locus (upper panel) and of the Pold1 domain structure (lower panel). The G2815T nucleotide change (red arrow) was located in exon 23. The corresponding D939Y amino acid substitution was located in the DNA polymerase domain close to the C-terminal domain (CTD); exo: exonuclease domain. (B) Multiple alignments of orthologous Pold1 amino acid sequences around D939Y among different eukaryotic organisms. The mutated aspartate residue was highly conserved. (C) Pold1 expression level in wildtype (left lane) and *tyrn* (right lane) embryos at E8.5 shown by Western blot. (D) Complementation test crossing strategy (upper panel). Wildtype and *tyrn/tm1b* embryos were acquired at E7.5 from the complementation test (lower panel). *tyrn/tm1b* embryos were not able to survive past E7.5. We did not remove the parietal yolk sac from the *tyrn*/*tm1b* embryo due to the challenge of dissecting the parietal yolk sac away from the fragile mutant embryo. (E) Crossing strategy to harvest homozygous mutants from heterozygous mice carrying the *tyrn* allele (upper panel). E7.5 wildtype and mutant embryos (lower panel). *n*=3 per genotype. Scale bar: 100 μm.

To identify the causative mutation for the *tyrn* phenotype, we collected both wildtype and *tyrn* mutant embryos at E8.5 and performed whole-exome sequencing (Jain et al., 2017). We found a G to T transversion at nucleotide position 2815 of the *Pold1* open reading frame that generated an aspartate to tyrosine substitution (D939Y) within the DNA polymerase domain (Fig. 2A)*.* The mutated aspartate residue is highly conserved across eukaryotic organisms from budding yeast to humans (Fig. 2B). Western-blot analysis showed a reduced level of Pold1 in *tyrn* mutant embryos (Fig. 2C). To confirm that *Pold1* is the causative gene underlying the *tyrn* mutant phenotype, we performed a complementation test using the *Pold1^tm1b^* null allele, derived from embryonic stem cells carrying a ‘knockout-first’ *tm1a* allele (Skarnes et al., 2011) (Fig. 2D, Fig. S1B). No *Pold1^tm1b/tm1b^* embryos were recovered at post-implantation stages, consistent with the phenotype previously reported for *Pold1*-null mutants (Uchimura et al., 2009). The *Pold1^tyrn/tm1b^* embryos produced from a *Pold1^tm1b/+^* and *tyrn/+* cross failed to survive past ∼E7.5 (Fig. 2D), demonstrating that the *tm1b* and *tyrn* mutations failed to complement. Moreover, the phenotype displayed by *Pold1^tyrn/tm1b^* embryos was more severe than that of the *Pold1^tyrn/tyrn^* embryos (Fig. 2E), but milder than that of *Pold1^tm1b/tm1b^*-null mutants. These findings indicate that perturbation of *Pold1* function is responsible for the phenotypes observed in *tyrn* mutants and that *Pold1^tyrn^* is a hypomorphic allele.

### The *tyrn* mutation impairs DNA synthesis and cell proliferation

**Fig. 3.**
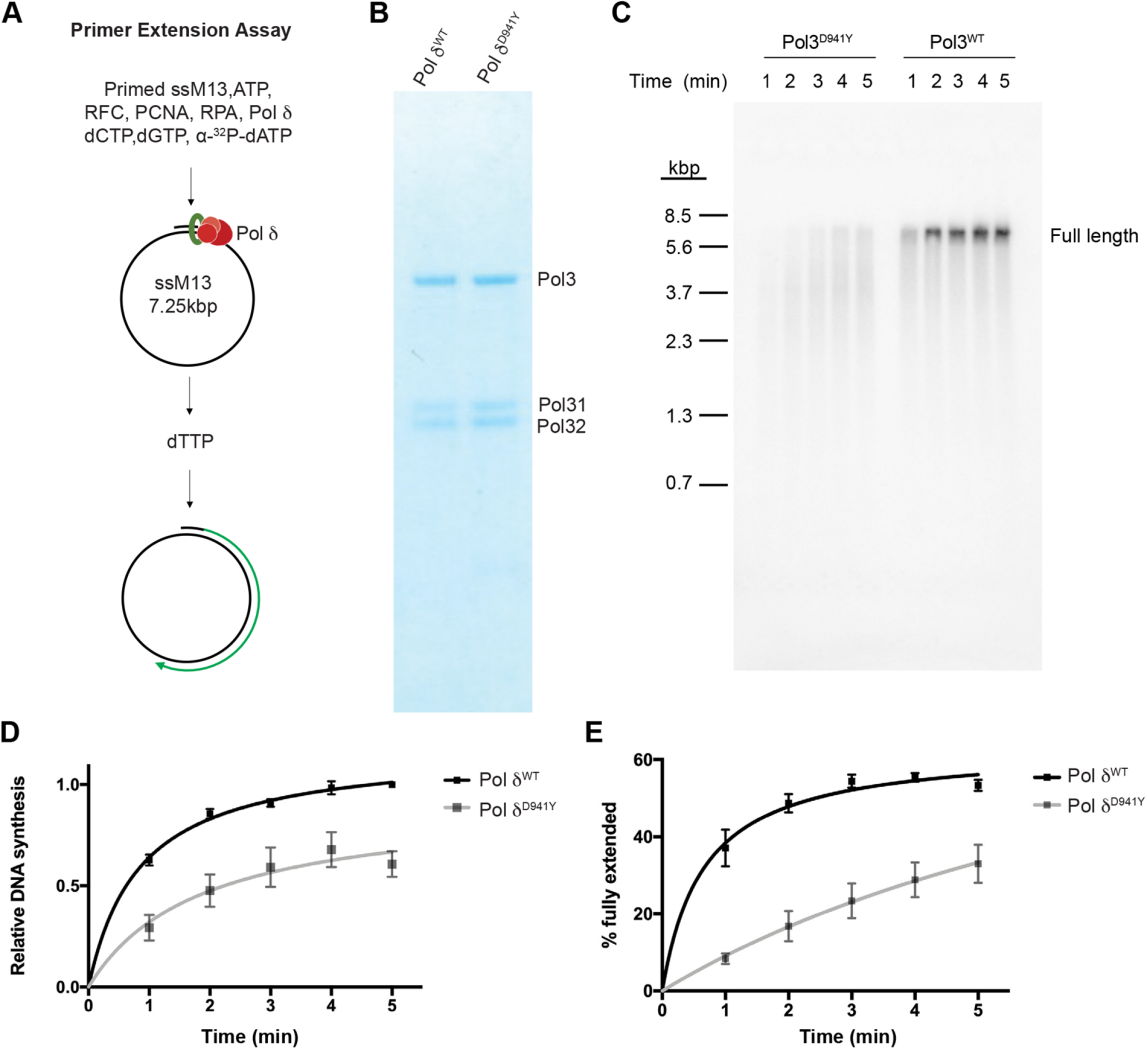
**The effects of the *D941Y* mutation in *Pol3* on DNA synthesis.** (A) Workflow of the *in vitro* primer extension assay. (B) Denatured protein gel showing the overexpressed yeast Pol δ^WT^ and Pol δ^D941Y^. The Pol3^WT^ and Pol3^D941Y^ correspond to mouse Pold1^WT^ and Pold1^D939Y^, respectively. The Pol31 and Pol32 are the associated subunits in Pol δ corresponding to Pold2 and Pold3 in mouse. (C) *In vitro* primer extension assay showing reduction of DNA synthesis efficiency of Pol3^D941Y^, the yeast orthologue which harbors the corresponding D939Y mutation identified in mouse. (D) Nonlinear-fitted curves of total amount of newly synthesized DNAs at different time points. The mean is represented by black squares (Pol δ^WT^) and grey squares (Pol δ^D941Y^). (E) Nonlinear-fitted curves of the percentage of full-length circular DNAs among total products. The mean is represented by black squares (Pol δ^WT^) and grey squares (Pol δ^D941Y^). Error bars represent s.e.m. For all time points in (D) and (E), *n*=3 per genotype. Multiple Student's *t*-test, two-tailed, *P*<0.05.

Based on the published cryo-electron microscopy structure of the human Pol δ (Lancey et al., 2020), the aspartate residue mutated in *tyrn* embryos resides in the thumb domain of the C-terminal DNA polymerase domain of POLD1 (Fig. 2A, Fig. S2A-C). Because of the important role of the thumb domain in stabilizing Pol δ at the primer–template junction during DNA synthesis (Jain et al., 2018), we hypothesised that the *tyrn* mutation may impair and reduce the DNA synthesis activity of Pol δ. We used a primer extension assay and mouse EdU labelling to test the Pol δ polymerase activity *in vitro* and *in vivo*, respectively. For the primer extension assay, we modelled the mouse Pold1 D939Y missense mutation in budding yeast Pol δ by introducing the equivalent mutation, D941Y, in the yeast catalytic subunit, Pol3 (Devbhandari and Remus, 2020) (Fig. 3A). We purified wildtype Pol δ or Pol δ^D941Y^ (with Pol3^D941Y^) after overexpression in budding yeast (Fig. 3B). Analysis of the DNA synthesis products by denaturing gel analysis reveals that both overall DNA synthesis and the level of full-length DNA products were significantly reduced in the presence of Pol3^D941Y^ when compared to the wildtype Pol δ (Fig. 3C-E). These data demonstrate that the D941Y substitution in Pol3, and by extension the corresponding D939Y substitution in Pold1, impairs the DNA polymerase activity of Pol δ, possibly by decreasing its processivity.

**Fig. 4.**
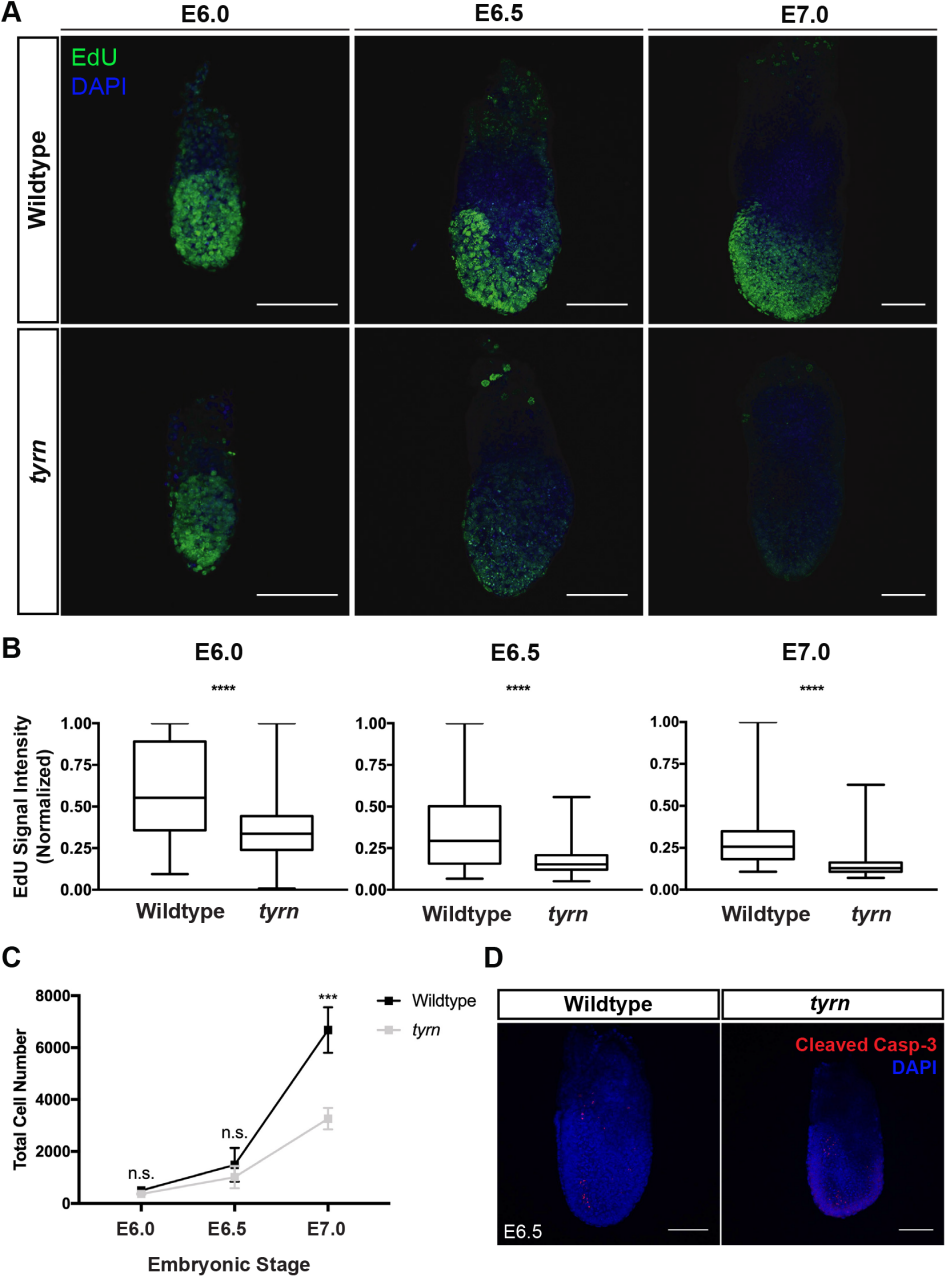
**Analysis of DNA synthesis and cell proliferation in *tyrn* mutants.** (A) EdU incorporation levels in wildtype and mutant embryos at E6.0, E6.5 and E7.0. Green: EdU, Blue: DAPI. *n*=3 embryos per genotype. Scale bar: 100 μm. (B) Quantification of EdU signal intensity by box whisker plots. EdU signals of the embryos at the same stage were normalised to the maximum intensity in that stage. Total embryos quantified per genotype: *n*=3. Unpaired Student's *t*-test, two-tailed. The four asterisks indicate *P*<0.0001. In all box plots, the median is represented by the horizontal dividing line; the top and bottom of the box represent, respectively, the seventy-fifth and twenty-fifth percentile, with the whiskers indicating the maximum and minimum points. (C) Curves of total cell number in wildtype and *tyrn* embryos at E6.0, E6.5 and E7.0; *n*=3 embryos per genotype per stage. Unpaired Student's *t*-test, two-tailed, *P*<0.001. Within the curve, the mean is represented by the black (wildtype) and grey (*tyrn*) squares. Error bars represent s.d. (D) Immunofluorescence staining of Cleaved Caspase-3 in wildtype and mutant embryos at E6.5; *n*=3 per genotype. Scale bar: 50 μm.

To test if DNA synthesis is affected in *tyrn* embryos, we performed *in vivo* EdU labelling at E6.0, E6.5, and E7.0, the window of time during which the size difference between wildtype and *tyrn* embryos emerges. We observed a significant reduction in EdU incorporation in *tyrn* mutants compared to wildtype embryos at all stages (Fig. 4A-B). Both wildtype and *tyrn* embryos showed a substantial increase in cell number from E6.0 to E7.0, but *tyrn* embryos exhibited a slower growth rate starting from E6.5; by E7.0 *tyrn* embryos had significantly lower numbers of cells compared to wildtype embryos (Fig. 4C). The reduction in cell number is not due to increased cell death; based on cleaved Caspase-3 staining, levels of cell apoptosis were similar between wildtype and *tyrn* embryos during this period (Fig. 4D). However, *tyrn* embryos showed increased cell apoptosis at around E7.5, with apoptotic cells concentrated at the distal tip, the prospective location of the abnormal small head at E8.5 (Fig. S3). Taken together, we conclude that the D939Y missense mutation impairs Pold1 polymerase activity, which, together with the reduced Pold1 protein expression in *tyrn* embryos, impedes cell proliferation and leads to a reduction in embryo size during gastrulation.

### The *tyrn* mutation does not affect anterior visceral endoderm positioning

**Fig. 5.**
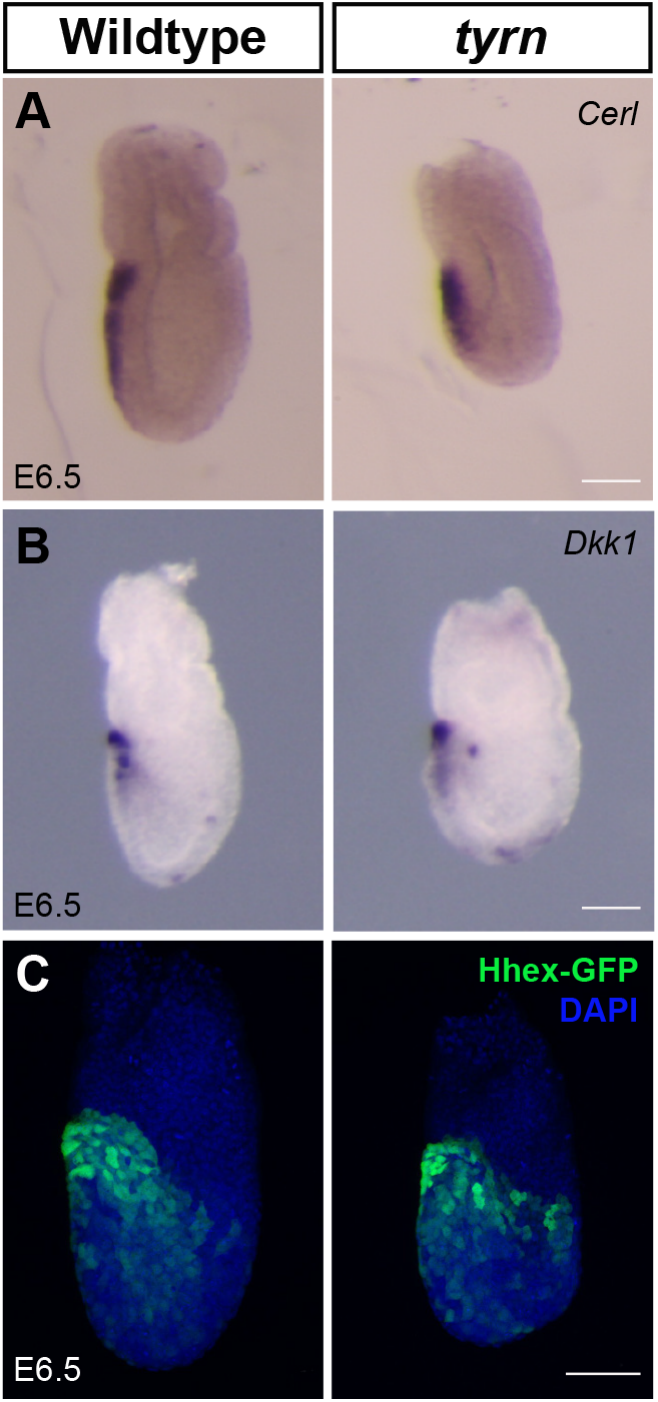
**Position of anterior visceral endoderm in *tyrn* mutants.** *Cerl* (A) and *Dkk1* (B) were expressed in anterior visceral endoderm (AVE) in both wildtype and *tyrn* embryos at E6.5. (C) Hhex-GFP was expressed in AVE in both wildtype and *tyrn* embryos at E6.5. *n*=3 embryos per genotype. Scale bar: 50 μm.

The mispositioned head, and the abnormal morphology of E8.5 *tyrn* mutants, indicated that the A–P axis, although established, was shifted in orientation along the proximal–distal axis. Proper formation of the A–P axis depends on the anterior migration of a morphologically distinct population of VE cells from their position at the distal tip of the E5.5 embryo (Rivera-Pérez et al., 2003); by E5.75-E6.0 this cell population will reach the anterior-epiblast–extraembryonic-ectoderm boundary and form the anterior visceral endoderm (AVE) (Srinivas et al., 2004). Multiple studies have shown that defects in AVE migration can lead to abnormal positioning of the A–P axis and mislocalised head phenotypes (Acampora et al., 2009; Clements et al., 2011; Kojima et al., 2014; Lu et al., 2001; Martinez-Barbera et al., 2000; Rossant and Tam, 2009). Therefore, we asked whether the AVE resided in its normal anterior position in *tyrn* mutants at E6.5. At this stage, the *tyrn* mutants were morphologically indistinguishable from wildtype littermates. WISH showed that *tyrn* mutants expressed two archetypal AVE markers, *Dkk1* (a Wnt antagonist) and *Cerl* (a Nodal antagonist), in a pattern comparable to that of wildtype embryos (Fig. 5A,B). We validated these findings by crossing in the Hhex-GFP transgene reporter to fluorescently label AVE cells in *tyrn* mutants (Rodriguez et al., 2001). Consistent with the *Dkk1* and *Cer1 in situ* hybridisation results, we detected the Hhex-GFP expressing AVE cells at the anterior boundary between the embryonic and extraembryonic regions in *tyrn* embryos at E6.5 (Fig. 5C). These results indicate that AVE migration and A–P axis establishment proceed normally in pre-gastrulation stage *tyrn* mutants.

### The *tyrn* mutation affects primitive streak extension and head position at E7.5

**Fig. 6.**
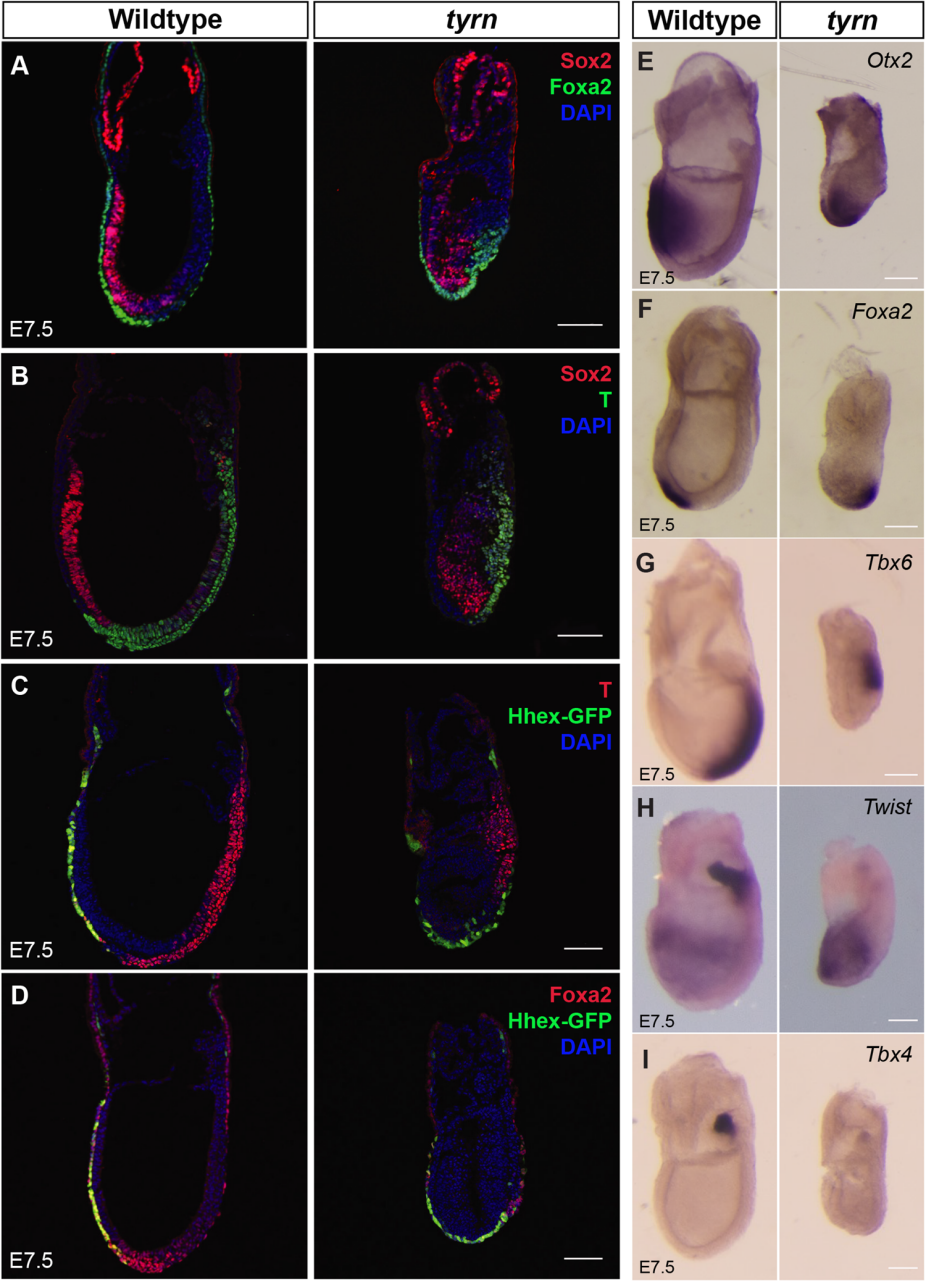
**Characterisation of primitive streak extension in *tyrn* mutants at E7.5.** Co-staining of Sox2 with Foxa2 (A) or with T (B). Co-localisation of Hhex-GFP with T (C) or Foxa2 (D). The anterior marker Sox2 was strongly expressed at the distal tip in mutants. Sox2 was also expressed in chorionic ectoderm in both wildtype and mutant embryos. T-staining found a shortened primitive streak in *tyrn* mutants; based on staining for Hhex-GFP and Foxa2, AME and ADE cells emerged from the midpoint of posterior side of *tyrn* mutants, in contrast to the distal/anterior region of wildtype embryos. (E) *In situ* hybridisation staining of *Otx2* showed that *Otx2* expression was restricted distally in *tyrn* rather than anteriorly, as seen in wildtype embryos. (F) *Foxa2* labels axial mesoderm and definitive endoderm cells in both wildtype and *tyrn* embryos. (G) In wildtype embryos, the paraxial mesoderm marker *Tbx6* was expressed along the posterior side down to the distal tip. In *tyrn* mutants, *Tbx6* expression was restricted to the posterior side of the embryo. (H) In wildtype embryos, *Twist* expression labelled anterior mesodermal precursors to cranial mesenchyme and extraembryonic mesoderm of the allantois. In *tyrn* embryos, *Twist* was not expressed in allantois. (I) *Tbx4* expression in allantois was strong in wildtype embryos but not detected in mutant embryos at E7.5. *n*=3 embryos per genotype. Scale bar: 100 μm.

At E6.5, the AVE resided at the expected location in *tyrn* mutants; yet the position of the *tyrn* embryo's anterior region was shifted toward the distal tip at E7.5. We examined the expression of multiple anterior-specific markers to visualise the organisation of the anterior region in both wildtype and *tyrn* mutant embryos at E7.5. As depicted in Fig. 6A-B, wildtype embryos expressed Sox2 uniformly throughout the anterior epiblast, whereas the *tyrn* mutants expressed Sox2 in a discontinuous pattern, with more intense staining in the distal epiblast than in the proximal/anterior region. In the wildtype E7.5 embryo in the left panels of Fig. 6C,D, the Hhex-GFP transgene labelled anterior definitive endoderm (ADE) as well as remaining AVE cells. In contrast, Hhex-GFP expression was evident predominantly in the distal region of the E7.5 *tyrn* mutant, with no obvious A–P asymmetry (Fig. 6C,D, right panels). Similarly, cells expressing *Otx2*, a head organiser marker, lay distally in *tyrn* embryos compared to their anterior position in wildtype embryos (Fig. 6E).

The AVE serves as a transient anterior signalling centre at ∼E6.5. As gastrulation progresses, AVE cells disperse into the extraembryonic–embryonic boundary and the anterior portion of the extraembryonic yolk sac (Lawson and Pedersen, 1987; Rivera-Pérez et al., 2003; Shimono and Behringer, 2003; Tam and Loebel, 2007). Meanwhile, the ADE and axial mesoderm (AME), both of which strongly express Nodal and Wnt antagonists (Arnold and Robertson, 2009), gradually migrate anteriorly and become new sources of anterior signalling (Arkell and Tam, 2012). We hypothesised that the abnormal orientation of the A–P axis might reflect the distal positioning of AME and ADE. This hypothesis is consistent with the aforementioned aberrant expression pattern of Hhex-GFP, which labels both ADE and AVE cells. Furthermore, Fig. 6A,D and F examined the expression of *Foxa2*, a marker of AME and ADE, in wildtype and *tyrn* embryos at E7.5. Emerging *Foxa2*-expressing ADE and AME cells resided in the distal anterior region in wildtype embryos, but these cells were observed more posteriorly in *tyrn* mutants.

The aberrant posterior positioning of ADE and AME, both derivatives of the anterior primitive streak (APS) (Arnold and Robertson, 2009; Lawson, 1999; Lawson et al., 1991), suggested that primitive streak extension was defective in *tyrn* mutants. To compare the organisation of the primitive streak between wildtype and *tyrn* embryos, we performed section immunofluorescence for T (Brachyury), a marker of nascent mesoderm (including AME) emanating from the primitive streak (Fig. 6B,C). In E7.5 wildtype embryos, T staining showed that the primitive streak had extended to the distal tip and generated AME derivatives of the anterior primitive streak. In contrast, T staining of E7.5 *tyrn* embryos indicated that the primitive streak had extended only to the midpoint of the posterior side (Fig. 6B,C). Also pointing to impaired primitive streak elongation in *tyrn* embryos, WISH detected irregularities in the production and positioning of paraxial and extraembryonic mesoderm. Whereas expression of *Tbx6,* a marker of paraxial mesoderm, was found along the posterior side of the wildtype embryo, down to the distal tip, it was restricted to the posterior–proximal region in *tyrn*, consistent with a short primitive streak (Fig. 6G). *Twist* expression labels two distinct mesodermal populations at E7.5: anterior mesoderm precursors to cranial mesenchyme and extraembryonic mesoderm of the allantois (Bildsoe et al., 2009). Of note, the E7.5 *tyrn* mutant generated *Twist*-expressing anterior mesoderm but lacked *Twist*-expressing cells of the allantois (Fig. 6H). The *tyrn* embryos also expressed greatly reduced levels of *Tbx4*, another marker of allantoic mesoderm (Fig. 6I). These findings indicate that *tyrn* mutants have very few extraembryonic mesoderm cells inside the allantois. Taken together, these results suggest that impaired extension of the primitive streak in *tyrn* embryos causes defects in multiple streak derivatives, including a severe deficiency of allantoic mesoderm and aberrantly positioned AME and ADE in the distal region of the E7.5 *tyrn* embryo. The development of the head distally, rather than anteriorly, in *tyrn* mutants, is potentially a consequence of impaired primitive streak extension as well. In wildtype embryos, the AME of the notochordal plate lies posterior to the prechordal plate and head process, midline mesodermal populations that provide signals governing the growth and patterning of neuroectoderm (Balmer et al., 2016; Costello et al., 2015). If their placement is coordinated with the position of the AME and ADE, then the prechordal plate and head process likely reside in an abnormal distal position, where they direct the formation of the head in *tyrn* mutants.

## DISCUSSION

**Fig. 7.**
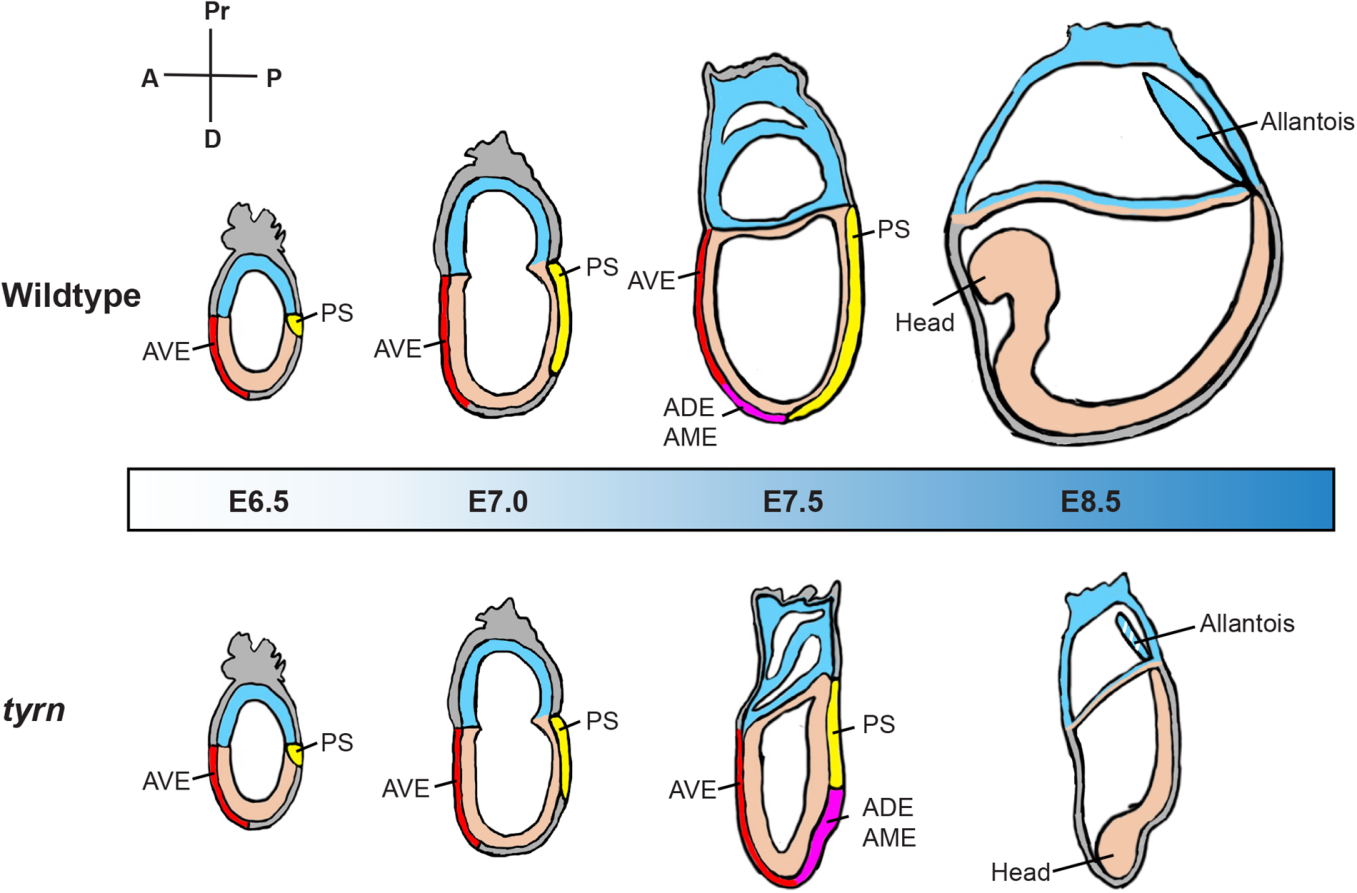
**Schematic illustration of the developmental progression of wildtype and *tyrn* embryos between E6.5 and E8.5.** At E6.5, wildtype and *tyrn* embryos are morphologically indistinguishable. AVE (red) is properly localised at the normal anterior region in both wildtype and mutant embryos. Gastrulation is initiated and a primitive streak (yellow) is formed. At E7.0. primitive streak elongation in *tyrn* mutants shows a slight delay compared to wildtype embryos, and there is a small though noticeable reduction in embryo size. In E7.5 wildtype embryos, the AME and ADE replace the role of AVE as the new signalling centre for head formation at the anterior side of the embryo. In *tyrn* mutants, there is reduced primitive streak extension, and the AME and ADE (magenta) appear at the midpoint of the posterior side instead of migrating across the distal tip of the embryos. In addition, the head forms at the distal tip with an abnormal morphology. At E8.5, wildtype embryos show a well extended A–P axis, whereas *tyrn* mutant embryos exhibit a small, siren-like morphology with the head pointing to the distal tip of the embryo. The white stripes indicate the reduction of extraembryonic mesoderm cells in the allantois.

The regulation of cell number plays an integral role in mouse embryo size determination during the pre-gastrulation stages. Earlier studies used embryo manipulation or pharmacological methods to alter cell numbers in pre- and early post-implantation mouse embryos. The findings showed, that prior to gastrulation, mouse embryos have the ability to sense and respond to an acute, global alteration in cell number and achieve normal size. Thus, robust size regulatory mechanisms must operate intrinsically in the mouse embryo to ensure proper size before the onset of gastrulation (Lewis and Rossant, 1982; Power and Tam, 1993). These classic studies raise many questions about when, in what cell populations, and how size-regulatory mechanisms function. Such questions are not amenable to approaches involving the global loss or gain of cells, especially for investigations during and after gastrulation. More suitable would be fine-tuned genetic methods that allow manipulation of candidate components of the size regulatory process over a defined time period. Here, we assessed the impact on embryo growth and morphogenesis of *tyrn*, an ENU-induced hypomorphic mutation that reduced levels of DNA synthesis at gastrulation stages. The *tyrn* mutation disrupted the polymerase function of Pold1, causing reduced cell proliferation *in vivo*, starting at ∼E6.0. *tyrn* embryos did not merely exhibit a wildtype-like morphology with a proportionally reduced size, or a random shape with no underlying logic. Instead, they showed a siren-like morphology, with the head located near the distal tip rather than in the anterior region, as seen in wildtype embryos. Phenotypic analyses performed at stages between E6.5 and E7.5 revealed that defective extension of the primitive streak likely underlies the mispositioned head. The AME and ADE produced by the short primitive streak resided in a distal position, causing the A–P axis to misalign with the proximal–distal axis (Fig. 7). We propose that in the *tyrn* mutant, decreased rates of DNA synthesis resulted in reduced levels of cell proliferation during gastrulation, which, in turn, disrupted the coordination of embryo growth with lineage specification and tissue morphogenesis. Therefore, the abnormal orientation of the *tyrn* mutant inside the decidua highlights the pivotal importance of the mechanisms orchestrating embryo growth with cell differentiation and movement during gastrulation.

Our study also suggests that reduced levels of cell proliferation during gastrulation perturb allocation of mesoderm lineages. Primitive streak formation initiates at the posterior–proximal pole where high levels of BMP, FGF, Wnt and Nodal signals are present (Brennan et al., 2001; Ciruna and Rossant, 2001; Conlon et al., 1994; Huelsken et al., 2000; Mishina et al., 1995). As the primitive streak elongates and extends to the distal tip, epiblast cells move into the streak and undergo EMT to form the mesoderm layer. The time and site of ingression through the streak determines the cell fate of mesoderm derivatives (Vincent et al., 2003; Winnier et al., 1995). Epiblast cell proliferation drives streak extension by providing a continuous supply of cells to populate the streak. In addition, normal rates of cell proliferation guarantee coordination of embryo size expansion with the formation of signal gradients; thus, exposing cells along the streak to different signal combinations and enabling them to contribute to different mesoderm subtypes. Since the primitive streak failed to fully extend in *tyrn* mutants, reduced cell proliferation likely disrupted such coordination. We found that *tyrn* mutants generated axial and paraxial mesoderm subtypes but contained them at a more posterior position. While the greatly reduced allantoic mesoderm may result from an altered posterior signalling environment, it could also reflect a general developmental delay, as allantoic mesoderm normally arises late during gastrulation, after the streak has reached the distal tip. Another possibility is that the extraembryonic mesoderm of the allantois originates from a highly proliferative cell population that is more sensitive to the inhibition of DNA synthesis. These questions may be addressed in the future through the perturbation of embryonic cell numbers in specific cell populations during gastrulation.

Cell number regulation occurs throughout different stages of embryonic development. The effects of cell number perturbation on tissue and organ size and morphogenesis can vary greatly based on developmental stage and tissue or organ type. The mandibular hypoplasia, deafness, progeroid and lipodystrophy (MDPL) syndrome, a multisystem disorder, has been associated with a heterozygous in-frame deletion of Ser605 that causes loss of POLD1 polymerase activity (Elouej et al., 2017; Fiorillo et al., 2018; Sasaki et al., 2018; Weedon et al., 2013). The reported patients presented growth retardation, sensorineural deafness, loss of subcutaneous adipose tissue and insulin resistance, indicating that decreased POLD1 activity exerts organ-specific effects. Other studies have also identified organ-specific responses to the loss of tissue-specific progenitors. Removing the embryonic limb field in amphibians or chickens does not affect the final limb size since the limb is capable of robust compensatory proliferation (Holder, 1981; Summerbell, 1981), and similar compensatory growth has been observed for the developing liver progenitors (Bort et al., 2006). In contrast, the final pancreas size is determined by the initial pancreatic progenitor pool and there is a lack of significant compensatory growth (Stanger et al., 2007). These studies demonstrate the importance of studying cell number regulation in diverse tissue and organ types at different stages of embryonic development. Our findings underscore the power of unbiased phenotype-based forward genetic screening approaches and highlight the value of using ENU-induced hypomorphic alleles to uncover new biological functions of well-characterised genes such as *Pold1*. The developmental biology field may benefit from revisiting unsolved questions from the ‘forgotten classics’ on embryo size regulation (https://thenode.biologists.com/forgotten-classics-regulating-size-mouse-embryo/research/) (Buehr and McLaren, 1974; Lewis and Rossant, 1982; Snow and Tam, 1979) by integrating newer methods and tools. For instance, one may use carefully chosen genetic models coupled with modern developmental biology techniques such as live imaging and single-cell genomics to investigate this important topic in diverse developmental contexts beyond the early stages of embryogenesis.

## MATERIALS AND METHODS

### Mouse strains (*Mus musculus*)

The parental JM8.N4 mouse ES cell strain (strain origin: C57BL/6N, male, black coat, non-agouti, MGI ID: 4431772) (Skarnes et al., 2011) carrying one *Pold1^tm1a(EUCOMM)Wtsi^* (*Pold1^tm1a^*) allele was imported from the European Mouse Mutant Cell Repository (EUMMCR). The selected Pold1-G09 ES cell clone passed the karyotype analysis performed by Molecular Cytogenetics Core Facility, Memorial Sloan Kettering Cancer Center (MSKCC). The Pold1-G09 ES cell clone was injected into the female B6(Cg)-Tyr^c-2J^/J (albino C57BL/6J, or B6-albino, non-agouti, The Jackson Laboratory) donor mice by MSKCC Mouse Genetics Core Facility. Fourteen out of the 19 pups alive were chimeric. Male chimeras were crossed with albino FVB/NJ (FVB, containing homozygous dominant *agouti* locus *A*/*A*) females to generate heterozygous offspring carrying the *Pold1^tm1a^* allele. Germline transmission was determined by the presence of agouti pups and genotyping for the presence of the *LacZ* cassette present in the *tm1a* allele. Mice carrying a *Pold1^tm1b^*-null allele were generated by crossing *Pold1^+/tm1a^* mice with *CAG-Cre* transgenic mice (The Jackson Laboratory) to remove the critical exons between exon 3 to exon 10. The *lacZ* cassette remained in the *Pold1^tm1b^* allele and was used for genotyping. The Hhex-GFP strain was a gift from Anna-Katerina Hadjantonakis (Developmental Biology Program, Sloan Kettering Institute) (Rodriguez et al., 2001). Mice that were 8-16 weeks old were used to generate E6.5 to E8.5 embryos. Analysis of the mutant phenotype was performed in the FVB or the mixed FVB-B6 genetic background. Mice were housed and bred under standard conditions in accordance with Institutional Animal Care and Use Committee (IACUC) guidelines. All experimental procedures were approved by the MSKCC IACUC.

### ENU allele isolation, sequencing and genotyping

The *tyrn* allele was generated by mouse ENU mutagenesis screens using C57BL/6J males and was identified based on its embryonic phenotype at E8.5, as previously described (García-García et al., 2005). To obtain genomic DNA, E8.5 *tyrn* mutant embryos were pooled into three sample groups and snap frozen on dry ice. Gentra Puregene kit (QIAGEN) was used for the genomic DNA extraction of *tyrn* mutant embryos. Whole-exome sequencing was performed at the MSKCC Integrated Genomics Operation. Exome capture was performed using the SureSelectXT kit (Agilent Technologies) and SureSelect Mouse All Exon baits (Agilent Technologies). An average of 100 million 75-bp paired reads were generated. Sequencing data analysis was performed using the methods described in (Jain et al., 2017). We examined homozygous exonic sequence variants shared among all pools of phenotypic embryos, but which were not identified in cohorts of wildtype embryo samples or separate lineages of ENU screen-derived embryos, and which were not present in a publicly available mouse genomic polymorphism database (dbSNP). Eleven potential phenotype-causing lesions were found in eight gene annotations, six nonsynonymous, of which *Pold1* was one: *Pold1*: NM_011131: exon23: c. G2815T: p.D939Y. After examining the known phenotypes of published alleles or functional annotations of these candidate genes, we re-examined non-exonic and heterozygous mutations and eliminated the read depth threshold to explore other possibilities. After going through the whole dataset (developed by Devanshi Jain from the Scott Keeney laboratory, Sloan Kettering Institute) (Jain et al., 2017) containing all our exome sequencing submissions over the years to catalogue universal variants we could eliminate, we confirmed that the sole candidate variant was in *Pold1.* The *tyrn* allele has a single G to T transversion in the 2815 nucleotide position of exon 23 in the *Pold1* coding sequence (G2815T), causing a nonsynonymous missense mutation in the amino acid position 939 resulting in the substitution of aspartic acid to tyrosine, D939Y. The G2815T mutation created a RsaI restriction site used for genotyping. Embryos displaying the *tyrn* morphological defect were homozygous for the G2815T mutation.

### Complementation test

*Pold1^tm1b/+^* females were crossed in timed matings with *tyrn/+* males to produce embryos. Embryos at E7.5 and E8.5 stages were harvested and grouped into wildtype and mutant cohorts based on phenotype. No *tyrn/tm1b* mutant embryos were recovered at E8.5 stage. The genotypes of E7.5 embryos corresponded with their morphological phenotypes, verifying that a lesion in *Pold1* was the causative mutation of the *tyrn* phenotype. Embryos with abnormal morphological phenotype genotyped *Pold1^tyrn/tm1b^*, whereas embryos with *Pold1^+/+^, Pold1^tyrn/+^ and Pold1^tm1b/+^* genotypes displayed wildtype phenotypes.

### Embryo harvesting and dissections

Pregnant FVB female mice bearing embryos at E6.5-10.5 stages were euthanised by cervical dislocation. Uteri were harvested based on an IACUC-approved mouse protocol. Embryos were dissected from decidua inside the uterus in cold 0.4% Bovine Serum Albumin (BSA) (Sigma-Aldrich) in phosphate-buffered saline (PBS) using fine forceps and Leica dissection scope. Embryos were fixed in 4% paraformaldehyde (PFA) on ice for at least 4 h and washed with PBS for 5 min three times at room temperature (RT). Fixed embryos were stored in PBS at 4°C for downstream experiments.

### *In situ* hybridisation

Fixed embryos were sequentially dehydrated in 25, 50%,75% methanol-DEPC PBS and 100% methanol and stored at −20°C. Embryos were sequentially rehydrated in 75%, 50%, and 25% methanol-DEPC PBS before experiments. *In situ* hybridisation was performed following the standard protocol (Eggenschwiler and Anderson, 2000). Briefly, after rehydration, embryos were incubated in 1 μg/ml Proteinase K/PBS solution for 3 to 7 min and hybridised in hybridisation solution with RNA probes at 70°C overnight. A series of washes in 2X SSC and MAB solutions were performed the next day. The embryos were incubated in 1:10000 anti-Digoxigenin antibody (Roche) in blocking buffer [1% blocking reagent (Roche), 10% heat-inactivated goat serum, 0.1% Tween-20 in PBS] overnight and washed extensively in PBS+0.1% Tween-20+0.1% BSA. Embryos were incubated in BM purple solution (Roche) at RT protected from light until the purple colour was developed.

### EdU labelling

EdU (5-ethynyl-2′-deoxyuridine) powder (Invitrogen) was dissolved in sterile PBS into a working concentration of 2.5 mg/ml. Mice were weighed and injected with EdU solution (25 mg/kg) intraperitoneally. Embryos were harvested 2 h after injection and fixed in 4% PFA overnight. Fixed embryos were washed with PBS+3% BSA twice and then permeabilised in PBS+0.5%Triton X-100 at RT for 20 min. Embryos were washed in PBS+3% BSA after permeabilisation. Embryos were incubated with the reaction cocktail made from Click-iT™ EdU Cell Proliferation Kit for Imaging, Alexa Fluor 637 dye kit (Invitrogen) at RT for 30 min, protected from light. The cocktail was removed after incubation and embryos were washed in PBS+3% BSA twice.

### Immunofluorescence and confocal microscopy

Fixed embryos were stored in PBS at 4°C before use. Embryos for cryosection were dehydrated in 30% Sucrose-PBS at 4°C overnight. Embryos were embedded in Tissue-Tek O.C.T Compound (Sakura Finetek) and frozen on smashed dry ice immediately after embedding. Embedded embryos were stored at −80°C before use. Embryos were sectioned in 10 μm using Leica CM1520 Cryostat. Section slides were stored at −80°C before use. For whole-mount staining embryos, embryos were permeabilised with PBS+0.5% Triton X-100 for 1 h at RT. For section staining, slides were dried for 30 min and incubated in blocking buffer [0.1% TritonX-100, 1% heat-inactivated donkey serum (Gemini Bio) in PBS] for 1 h at RT. Primary antibodies were diluted with the optimised dilution ratio in blocking buffer: Foxa2 (Abcam, catalogue number ab108422, RRID:AB_11157157,1:300), T (Cell Signaling Technology, catalogue number 81694, RRID:AB_2799983,1:400), Cleaved Caspase-3 (Cell Signaling Technology, catalogue number 8202, RRID:AB_1658166,1:300), Sox2 (R and D Systems, catalogue number AF2018, RRID:AB_355110,1:300). Embryos and slides were incubated with primary antibodies at 4°C overnight. Embryos and slides were washed with PBS for 10 min three times the next day. After that, section slides or embryos were incubated in the blocking buffer containing specific secondary antibodies (Invitrogen,1:500) and DAPI (1:1000) for 2 h at RT. For whole-mount staining embryos, embryos were washed in PBS for 5 min three times, incubated in FocusClear (CelExplorer.Co) for 20 min at RT, and protected from light. Embryos were mounted with MountClear (CelExplorer.Co) and stored at 4°C. For cryosection staining, slides were washed in PBS for 5 min three times. Slides were mounted with ProLong Gold Antifade Mountant (ThermoFisher Scientific) and stored at 4°C. Both embryos and sections were imaged using Leica SP8 inverted laser scanning confocal microscope. Confocal images were reconstructed using Fiji (ImageJ) open-source image processing software.

### Immunoblotting

E8.5 wildtype and *tyrn* embryos were harvested and pooled separately and stored at −80°C before experiments. Tissues were homogenised in cold lysis buffer [0.1% NP-40, 50 mM Tris-HCl (pH 7.2), 250 mM NaCl, 2 mM EDTA, phosphatase inhibitor mixture I and II (Calbiochem) and one tablet of Minicomplete (Roche) per 10 ml] on ice. Lysate was left on the shaker at 4°C for another 30 min. Lysate was centrifuged at maximum speed (12,000 rpm) to collect supernatant. Protein concentration was determined through BSA-based protein assays using Quick Start Bradford 1X Dye Reagent (BIORAD) and adjusted to a final concentration of 2 μg/μl. Samples were mixed 1:1 with 2X SDS loading buffer for denaturing at 95°C for 5 min. Then, samples were loaded in equal amounts onto 8% SDS-PAGE gels and run for 2 h under 150 V in 1X SDS buffer at RT. Proteins were transferred to PVDF membranes under 15 V overnight at 4°C. Membranes were incubated in blocking buffer (TBST+5% BSA) for 1 h at RT and incubated with primary antibodies in blocking buffer at 4°C overnight: POLD1 (Abcam, catalogue number ab168827,1:500); GAPDH (Santa Cruz Biotechnology, catalogue number sc-32233, RRID: AB_627679, 1:1000). Membranes were washed with TBST and incubated with specific secondary antibodies for 1 h at RT. Finally, membranes were washed with TBST and incubated with Pierce ECL Western Blotting Substrate (ThermoFisher Scientific) for 5 min and were film-exposed to show target bands.

### Primer extension assay

Primer extension reactions were performed at 30°C in polymerisation buffer (25 mM Tris-HCl pH 7.5, 8 mM Magnesium Acetate, 5 mM Potassium Glutamate, 5% Glycerol). Purified proteins used in the primer extension assays were purified as previously described (Devbhandari and Remus, 2020). DNA template for the assay was generated by annealing a primer (5′-CCCAGTCACGACGTTGTAAAACG-3′) to M13mp18 single-stranded DNA (New England Biolabs, N4040S). The assay was initiated by incubation of 1 nM of DNA template with 1mM ATP, 1mM DTT, 80 μM dATP, 80 μM dGTP, 80 μM dCTP and 400 nM of RPA for 5 min. PCNA and RFC were then added to 70 nM and 4 nM, respectively, and incubation was continued for 5 min. Then, 33 nM of α−^32^P-dATP (3000 Ci per mmol) and 4 nM of either Pol δ^WT^ or Pol δ^D941Y^ was added to the reaction, resulting in a primer extension by nine base pairs (due to lack of dTTP). After 5 min, 80 μM dTTP were added to the mix for synchronous primer extension. Equal volume aliquots of this reaction (18 μl) were removed from the master reaction (100 μl) at indicated times and stopped by adding EDTA and SDS to final concentrations of 40 mM and 0.25%, respectively. Products were fractionated on a 0.8% alkaline agarose gel (30 mM NaOH and 2 mM EDTA), dried and imaged using Typhoon FLA 7000. Quantification of the gel images was performed using ImageJ.

### Quantitation of total cells, EdU signal intensity, EdU-positive cells

E6.0, E6.5, and E7.0 whole-mount EdU-labeled embryos were imaged using Leica SP8 inverted laser scanning confocal microscope and confocal z stacks of embryos were generated. *tyrn* mutant and wildtype embryos of the same stage were imaged under the same conditions. For each stage, three embryos per genotype were imaged for quantification. Optical sections of each embryo were imported into Imaris (version 9.5, Oxford Instruments), and three-dimensional reconstitution along Z-axis was performed for data analysis. We used the spots function in Imaris to automatically segment all DAPI-positive cells (in both embryonic and extraembryonic tissues) to obtain a total cell count for each embryo. EdU signal intensity of each segmented cell was automatically measured, and the background signal was subtracted. Cells with EdU signal above 10 arbitrary units were considered EdU positive and counted as EdU positive cells by the software. We used Prism 9 (GraphPad) to perform normalisation of EdU signal intensity. At each stage, the EdU signal of each EdU positive cell was normalised to the maximum EdU signal intensity. Data points were presented as box plots to show the distribution of EdU signal intensity among EdU positive cell population. Within the box plot, the median is represented by the horizontal dividing line and the top and bottom of the box represent the 75th and 25th percentiles, with the whiskers indicating the maximum and minimum points. A two-tailed Student's *t-*test was performed to evaluate the significance of all measurements.

### Statistics and graphs

For all embryo imaging experiments, *n*=3. For primer extension assays, three biological replicates were performed. We used Prism 9 to perform two-tailed Student's *t-*tests to evaluate the significance of all measurements and generate box plots and non-linear fitted curves. For the structure of human DNA polymerase δ, the original structure was imported and processed in PyMOL (PyMOL Molecular Graphics System, Version 1.2r3pre. Schrödinger, LLC).

### Data availability

The structure of human DNA polymerase δ is from the Protein Data Bank (PDB). PDB DOI: 10.2210/pdb6TNY/pdb. EM Map EMD-10539: EMDB EMDataResource.

**Table:**
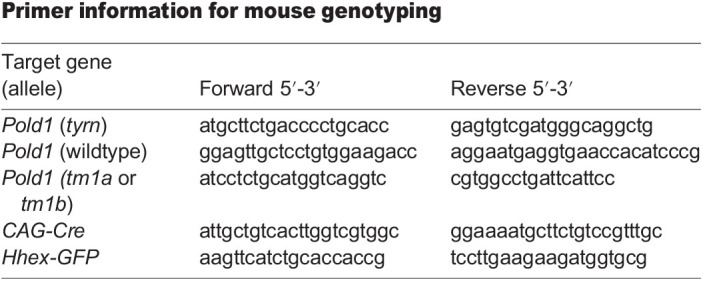


## Supplementary Material

Supplementary information
