# Supplementary material for: A hypomorphic mutation in Pold1 disrupts the coordination of embryo size expansion and morphogenesis during gastrulation
Source: Biol Open. 2022 Aug 8;11(8):bio059307. doi: 10.1242/bio.059307 (PMC9382117; doi:10.1242/bio.059307)
Supplement: Supplementary information [file biolopen-11-059307-s1.pdf]

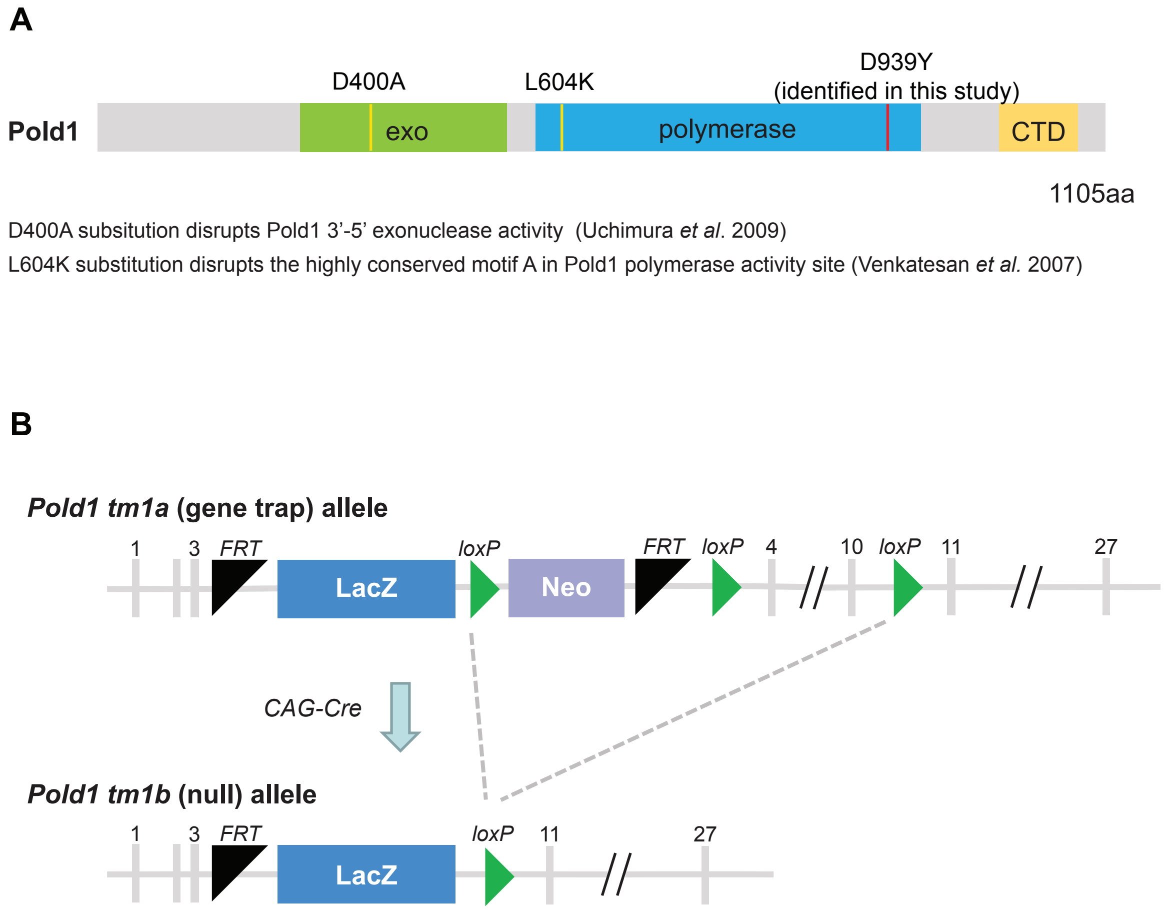

Fig. S1. Summary of *Pold1* mutant mice and crossing strategy for generating a *Pold1* null allele. (A) Schematic domain structure in mouse Pold1. The yellow bars represent mutations generated in previous genetic studies documented in the literature. The red bar denotes the missense mutation identified from the ENU mutagenesis screen in this study. (B) Schematic of the *Pold1 tm1a* (gene trap) allele and the *tm1b* (null) allele generated after CAG-Cre-mediated recombination. Exons are represented in grey vertical blocks.

Biology Open • Supplementary information

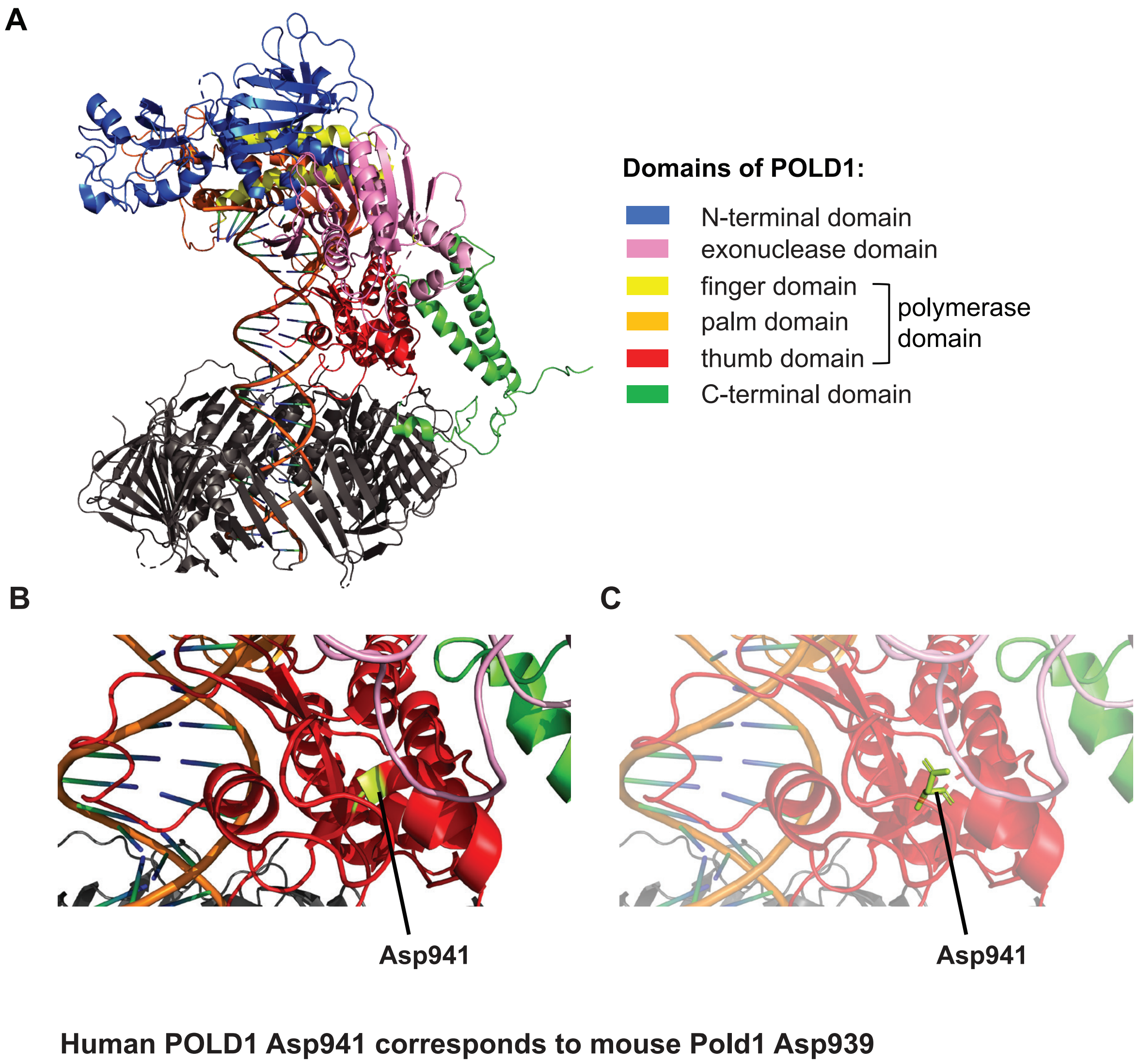

Fig. S2. Cryo-EM structure of human POLD1 associated with DNA duplex and PCNA. (A) Colored-coded domain structure of the POLD1-PCNA complex bound to DNA. (B) Zoomed-in image showing the POLD1 thumb domain (red). The mutated Asp941(D941) corresponding to mouse Asp939 (D939) residue is highlighted in lime. (C) Zoomed-in image showing the POLD1 thumb domain. The mutated Asp 941(D941) residue is shown as the lime stick. The protein structure information was extracted from protein data bank (PDB). Structure ID: 6TNY. PDB DOI: 10.2210/pdb6TNY/pdb. The original protein structure was adapted using PyMOL to convey information relevant to this study.

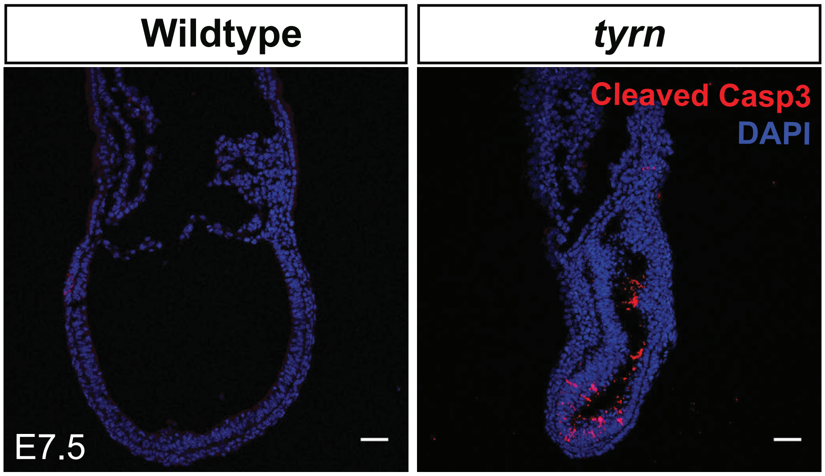

Fig. S3. Cell apoptosis in E7.5 embryos. Whole mount staining of E7.5 wildtype and *tyrn* embryos with Cleaved Caspase-3 antibodies (Red) for detection of apoptot-ic cells. DAPI (blue) stained nucleus of all cells. n = 3 per genotype. Scale Bar = 100 μm.

Biology Open • Supplementary information
